# Comparative Analysis of 30-Day Complications in Transgender Men Undergoing Different Routes of Hysterectomy: A Study Utilizing American College of Surgeons National Surgical Quality Improvement Program (ACS-NSQIP) Data

**DOI:** 10.7759/cureus.85118

**Published:** 2025-05-31

**Authors:** Bashar Hassan, James Antongiovanni, Dana Hazimeh, Alissa Haas, Fan Liang, Stephen Martin

**Affiliations:** 1 Center for Transgender and Gender Expansive Health, Johns Hopkins University, Baltimore, USA; 2 Medicine, Washington State University Elson S. Floyd College of Medicine, Spokane, USA; 3 Obstetrics and Gynecology, Johns Hopkins University, Baltimore, USA; 4 Plastic and Reconstructive Surgery, Southern Illinois University School of Medicine, Springfield, USA

**Keywords:** abdominal hysterectomy, gender-affirmation surgery, laparoscopic-assisted vaginal hysterectomy, laparoscopic hysterectomy, patient outcomes research, safety, surgical outcomes research, transgender health, transgender obstetrics and gynecology, vaginal hysterectomy

## Abstract

Introduction: Gender-affirming surgeries, including hysterectomy, have been shown to reduce distress and improve quality of life for transgender men. However, the safety and efficacy of different hysterectomy approaches in transgender men have not been extensively studied. Here, we aim to compare postoperative complications among the different hysterectomy approaches in transgender men.

Methods: This retrospective cohort study utilized the American College of Surgeons National Surgical Quality Improvement Program (ACS-NSQIP) database from 2012 to 2020 to compare 30-day postoperative complications across different routes of hysterectomy in transgender men. Transgender men who underwent hysterectomy were identified using International Classification of Diseases (ICD)-9 and ICD-10 codes for gender dysphoria. Routes of hysterectomy included laparoscopic hysterectomy (LH), laparoscopic-assisted vaginal hysterectomy (LAVH), abdominal hysterectomy (AH), and vaginal hysterectomy (VH). Descriptive statistics, bivariate analysis, and multivariate logistic regression were performed to evaluate the association between hysterectomy routes and complications.

Results: A total of 1333 transgender men were included. Most patients underwent LH (N=1068, 80.1%), followed by LAVH (N=169, 12.7%), VH (N=54, 4.1%), and AH (N=42, 3.2%). The overall 30-day incidence of complications was 6.5%, with surgical site infection and unplanned reoperation being the most common complications. LAVH was associated with a higher incidence of unplanned intubation, while AH had a higher incidence of bleeding requiring transfusion. After adjusting for baseline comorbidities, LAVH was associated with significantly greater odds of unplanned intubation compared with LH (adjusted odds ratio (aOR) 95% confidence interval (CI) 28.1 (2.3->100)).

Conclusion: This study provides important insights into the 30-day postoperative outcomes of different hysterectomy approaches in transgender men. LH appears to have the lowest incidence of complications, whereas LAVH and AH present higher risks for specific complications. These findings can aid in informed decision-making and surgical planning, ultimately improving care for transgender men undergoing hysterectomy.

## Introduction

The transgender and non-binary (TGNB) population is growing and becoming increasingly visible, leading to a significant increase in individuals seeking treatment for gender dysphoria [1]. Gender dysphoria, defined as psychological distress resulting from a discrepancy between one’s sex assigned at birth and one’s gender identity, can be addressed through both medical and surgical interventions [2]. Undergoing gender-affirming surgery has been shown to reduce psychological distress and improve the quality of life for TGNB patients [3]. Consequently, gender-affirming surgeries are being performed more frequently, with surgical options for transgender men including, but not limited to, hysterectomy, oophorectomy, vaginectomy, metoidioplasty, phalloplasty, and mastectomy [4]. A survey by the National Center for Transgender Equity in 2011 found that 21% of transgender men have had a hysterectomy, and almost 60% would like to have the procedure in the future [5].

By definition, a total hysterectomy involves the removal of the uterus and cervix. Hysterectomies can be performed using laparoscopic, laparoscopic-assisted vaginal, vaginal, and abdominal approaches. Laparoscopic hysterectomy (LH) is a minimally invasive approach involving small abdominal incisions for insertion of laparoscopic machinery and removal of the uterus and cervix through these abdominal incisions laparoscopically post-morcellation. Another minimally invasive technique is vaginal hysterectomy (VH) in which the uterus and cervix are removed through an incision made above the vagina. Laparoscopic-assisted vaginal hysterectomy (LAVH) combines laparoscopic and vaginal approaches by removing structures above the uterine artery laparoscopically with vaginal removal of remaining structures. Abdominal hysterectomy (AH) carries a higher incision burden requiring a large bikini-line abdominal incision for removal of the uterus and cervix [6]. The safety and efficacy of different hysterectomy approaches have been extensively compared in cisgender females [6-11], but similar studies have not been conducted in transgender men.

The aforementioned surgical approaches vary by indication. LAVH is often utilized when VH alone is challenging due to limited access or anatomical considerations. This approach is particularly beneficial when a patient desires a minimally invasive surgery, but the size or position of the uterus makes complete laparoscopic removal difficult [12]. On the other hand, AH is indicated when the uterus is significantly large, or when there is extensive pelvic pathology e.g., adhesions, making other approaches impractical. AH is also the preferred approach when there is a suspicion of gynecologic malignancy or when minimally invasive methods are contraindicated due to factors such as severe obesity, extensive previous abdominal surgeries, or inadequate laparoscopic visualization [13].

Hysterectomy for gender dysphoria is often performed without concurrent gynecologic indications. Because of this, previous studies have been concerned with the feasibility of hysterectomy in this population. Our literature review found no studies comparing the routes of hysterectomy in transgender men. The few existing studies on the outcomes of transgender patients undergoing hysterectomy are often limited by small sample sizes and comparisons to cisgender patients undergoing hysterectomy for gynecological disease [8,14,15]. Understanding the safest and most effective surgical route for hysterectomy in transgender men is essential to optimizing outcomes, minimizing complications, and guiding individualized surgical planning in gender-affirming care.

Hence, our objective was to compare the 30-day complications across the different routes of hysterectomy in transgender men utilizing the American College of Surgeons National Surgical Quality Improvement Program (ACS-NSQIP). We hypothesize that LH will be associated with the lowest incidence of complications, whereas AH will be associated with the highest incidence of complications. Our objective is to contribute to informed decision-making and surgical planning for hysterectomy in transgender men.

## Materials and methods

The ACS-NSQIP is an international initiative maintained by the American College of Surgeons. It involves over 750 voluntary-participating hospitals, which include critical access, community, and large academic teaching hospitals from the United States, Canada, and 11 other countries. The program reports various data variables, encompassing preoperative risk factors, intraoperative variables, and 30-day postoperative mortality and morbidity outcomes for patients undergoing surgical procedures. The de-identified data is then shared with participating institutes, facilitating research inquiries concerning surgical procedure outcomes and quality care enhancement [16].

This is a retrospective cohort study in which we queried the NSQIP database from 2012 to 2020 for TGNB individuals using ICD-9 and ICD-10 codes pertaining to gender dysphoria (Table 1). Transgender men who underwent hysterectomy as a principal procedure based on the current procedural terminology (CPT) codes outlined in Table 1 were selected. Excluded were patients who underwent unlisted laparoscopic procedures, hysterectomies with lymphadenectomy, and hysterectomies due to malignancy.

**Table 1:** ICD-9, ICD-10 and CPT Codes Used Acronyms – ICD: International Classification of Diseases; CPT: Current Procedural Terminology.

Description	ICD-9
Transsexualism with unspecified sexual history	302.50
Transsexualism with asexual history	302.51
Transsexualism with homosexual history	302.52
Transsexualism with heterosexual history	302.53
Gender identity disorder in children	302.6
Gender identity disorder in adolescents or adults	302.85
	ICD-10
Transsexualism	F64.0
Gender identity disorder in adults	F64.1
Gender identity disorder in children	F64.2
Other gender identity disorders	F64.8
Gender identity disorder, unspecified	F64.9
	CPT
Total abdominal hysterectomy (corpus and cervix), with or without removal of tube (s), with or without removal of ovary (s)	58150
Supracervical abdominal hysterectomy (subtotal hysterectomy), with or without removal of tube(s), with or without removal of ovary(s)	58180
Vaginal hysterectomy, for uterus 250 g or less	58260
Vaginal hysterectomy, for uterus 250 g or less	58262
Vaginal hysterectomy, for uterus 250 g or less	58263
Vaginal hysterectomy, for uterus 250 g or less.	58270
Vaginal hysterectomy, for uterus greater than 250 g.	58290
Vaginal hysterectomy, for uterus greater than 250 g	58291
Laparoscopy, surgical, supracervical hysterectomy, for uterus 250 g or less	58541
Laparoscopy, surgical, supracervical hysterectomy, for uterus 250 g or less	58542
Laparoscopy, surgical, supracervical hysterectomy, for uterus greater than 250 g	58544
Laparoscopy, surgical, with vaginal hysterectomy, for uterus 250 g or less	58550
Laparoscopy, surgical, with vaginal hysterectomy, for uterus 250 g or less	58552
Laparoscopy, surgical, with vaginal hysterectomy, for uterus greater than 250 g	58554
Laparoscopic total hysterectomy for uterus 250 g or less	58570
Laparoscopic total hysterectomy for uterus 250 g or less; with removal of tube(s) and/or ovary(s)	58571
Laparoscopy, surgical, with total hysterectomy, for uterus greater than 250 g	58572
Laparoscopy, surgical, with total hysterectomy, for uterus greater than 250 g	58573

Routes of hysterectomy were categorized into LH, LAVH, AH, and VH. Procedural outcomes were compared across these procedural routes. The primary outcome was the incidence of postoperative complications within 30 days following hysterectomy. The 30-day incidence of most postoperative complications was analyzed, namely unplanned reoperation, unplanned readmission, surgical site infection, wound disruption, pneumonia, pulmonary embolism, use of ventilator >48 hours, urinary tract infection, sepsis, septic shock, hospital stay >30 days, and Clostridium difficile infection. To assess the potential association between body mass index (BMI) and postoperative complications, the incidence of postoperative complications, as well as any postoperative complication, within 30 days was compared between different BMI categories (<30 kg/m2, 30-40 kg/m2, >40 kg/m2).

Descriptive statistics were calculated. Bivariate analysis and multivariate logistic regression were performed to evaluate the association between routes of hysterectomy and postoperative complications. Independent t-tests and Chi-squared test were used to compare continuous variables and proportions on bivariate analysis, respectively. LH was the reference group to which the other routes were compared in the multivariate logistic regression model. Statistical analysis was performed using IBM SPSS Statistics, Version 29 (IBM Corporation, Armonk, NY, USA). A P-value <0.05 was considered statistically significant.

## Results

**Figure 1:**
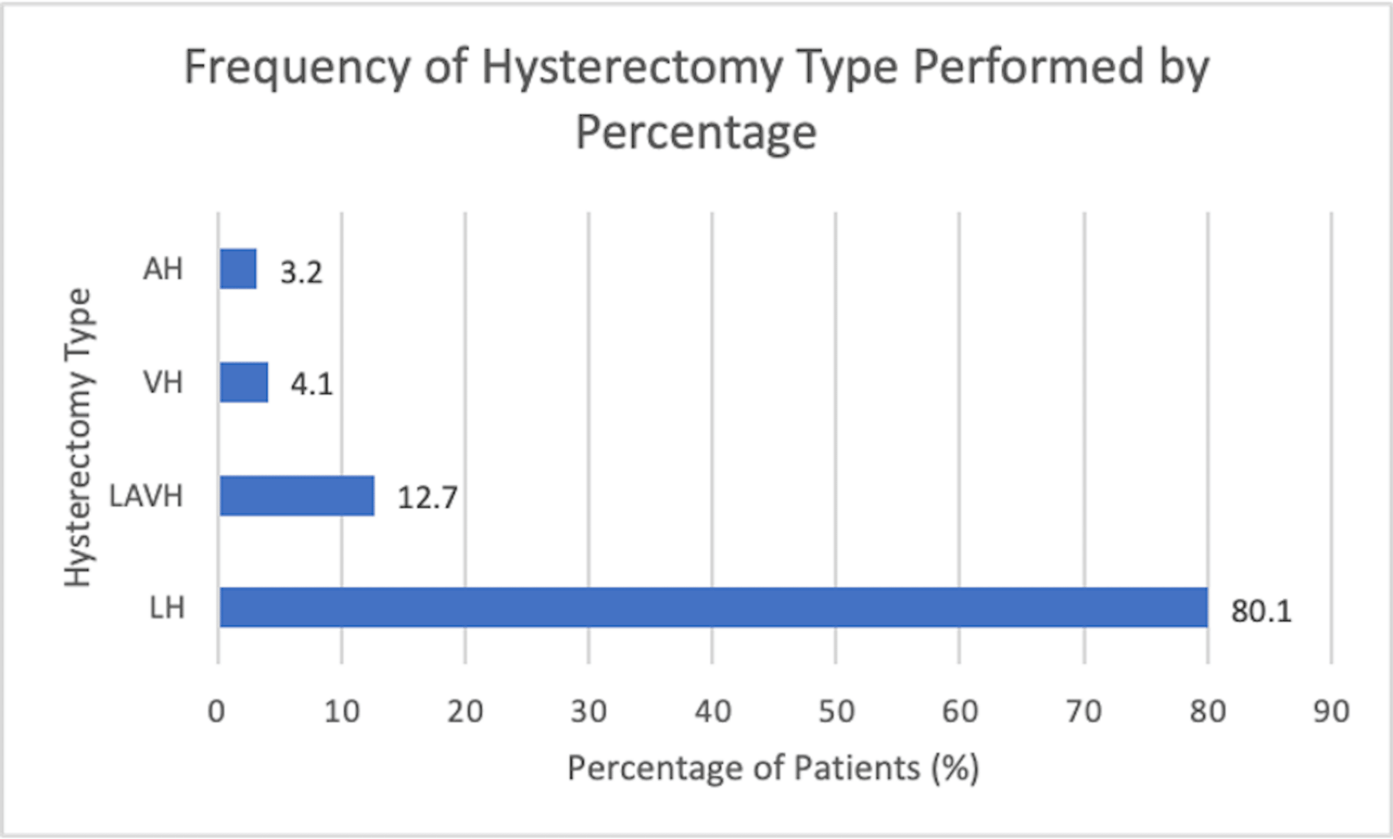
Frequency of hysterectomy type performed by percentage Acronyms – LH: laparoscopic hysterectomy; LAVH: laparoscopic-assisted vaginal hysterectomy; VH: vaginal hysterectomy; AH: abdominal hysterectomy. Bar graph illustrating the distribution of hysterectomy routes among patients in the study cohort. Most patients underwent laparoscopic hysterectomy (N=1068, 80.1%), followed by laparoscopic-assisted vaginal hysterectomy (N=169, 12.7%), vaginal hysterectomy (N=54, 4.1%), and abdominal hysterectomy (N=42, 3.2%).

A total of 1333 transgender men who underwent hysterectomy were included. Their mean (SD) age was 28.7 (8.1) years and mean (SD) BMI was 29.1 (7.2) kg/m2. The majority of patients were non-Hispanic Whites (N=841 (63.1%)) and underwent hysterectomy on an outpatient basis (N=1032 (77.4%)). Most patients underwent LH (N=1068 (80.1%)), followed by LAVH (N=169 (12.7%)), VH (N=54 (4.1%)), and AH (N=42 (3.2%)) (Figure 1).

Table 2 compares patient demographics and surgical characteristics across hysterectomy routes. Patients who underwent AH had a significantly higher average BMI (mean (SD) 32.2 (9.3)) compared with other routes. AH and LAVH were significantly more likely to be performed as inpatient procedures compared with LH and VH which were more likely to be performed as outpatient procedures (Table 2). The average (SD) total operating time was 122 (61) minutes and was not significantly different across routes. The average (SD) length of total hospital stay was 0.6 (1.7) days. The average length of stay for AH (>2 days) was significantly longer than that of other routes (<1 day). There was no significant difference in the prevalence of other demographics among the different routes (Table 2). However, patients who underwent AH were more likely to have American Society of Anesthesiologists (ASA) class 3 compared to those who underwent LH (N=9 (21.4%), N=87 (8.1%); P=0.011).

**Table 2:** The demographics and surgical characteristics of our study population compared across the different routes of hysterectomy. Acronyms – LH: laparoscopic hysterectomy; LAVH: laparoscopic-assisted vaginal hysterectomy; VH: vaginal hysterectomy; AH: abdominal hysterectomy; BMI: body mass index; ASA: American Society of Anesthesiologists; SD: standard deviation; IQR: interquartile range; F: F-value for ANOVA; χ: Chi-squared statistic. ANOVA is used to compare continuous variables. Chi-squared and Fisher’s exact tests are used to compare proportions. In particular, Fisher’s exact test is used when the count in a cell was less than 5. Subscript letters a and b indicate statistically significantly different proportions. P <0.05 is considered statistically significant.

Demographics and surgical characteristics			Overall (N=1333)	LH (N=1068)	LAVH (N=169)	VH (N=54)	AH (N=42)	Test statistic	P
Age, mean (SD), years			28.7 (8.1)	28.8 (8.1)	27.8 (6.9)	31.1 (9.7)	27.5 (6.3)	F=3	0.045
BMI, mean (SD), kg/m^2^			29.1 (7.2)	29.1 (7.1)_a_	28.2 (6.7)_a_	30.3 (7.9)_a,b_	32.2 (9.3)_b_	F=4	0.009
Total operating time, mean (SD), minutes			121.5 (60.5)	122.7 (60.1)	114.3 (56.5)	118.8 (62.9)	123.7 (80.5)	F=1	0.398
Length of total hospital stay, mean (SD), days			0.6 (7.7)	0.5 (1.7)_a_	0.7 (0.7)_a_	0.4 (0.6)_a_	2.1 (2.5)_b_	F=13	<0.001
Race/ethnicity, No. (%)		Hispanic	80 (6.0)	68 (6.4)	9 (5.3)	2 (3.7)	1 (2.4)	150	<0.001
		Non-Hispanic White	841 (63.1)	725 (67.9)_a_	57 (33.7)_b_	36 (66.7)_a_	23 (54.8)_a,b_		
		Non-Hispanic Black	99 (7.4)	79 (7.4)	11 (6.5)	5 (9.3)	4 (9.5)		
		Non-Hispanic, Asian	34 (2.6)	31 (2.9)_a,b_	0 (0.0)_a_	1 (1.9)_a,b_	2 (4.8)_b_		
		Non-Hispanic, American Indian	10 (0.8)	7 (0.7)	1 (0.6)	1 (1.9)	1 (2.4)		
		Others	269 (20.2)	158 (14.8)_a_	91 (53.8)_b_	9 (16.7)_a_	11 (26.2)_a_		
Inpatient/Outpatient Routes, No. (%)		Inpatient	301 (22.6)	173 (16.2)	89 (52.7)	9 (16.7)	30 (71.4)	χ=171	<0.001
		Outpatient	1032 (77.4)	895 (83.8)	80 (47.3)	45 (83.3)	12 (28.6)		
Surgical Specialty, No. (%)		General Surgery	6 (0.5)	3 (0.3)	2 (1.2)	0 (0.0)	1 (2.4)	25	0.033
		Gynecology	1316 (98.7)	1058 (99.1)	165 (97.6)	54 (100.0)	39 (92.9)		
		Obstetrics	5 (0.4)	4 (0.4)	1 (0.6)	0 (0.0)	0 (0.0)		
		Plastics	5 (0.4)	3 (0.3)	0 (0.0)	0 (0.0)	2 (4.8)		
Diabetes mellitus, No. (%)			29 (2.2)	28 (2.6)	0 (0.0)	1 (1.9)	0 (0.0)	6	0.121
Current Smoker, No. (%)			188 (14.1)	144 (13.5)	29 (17.2)	10 (18.5)	5 (11.9)	χ=3	0.439
Hypertension requiring medication, No. (%)			84 (6.3)	67 (6.3)	9 (5.3)	4 (7.4)	4 (9.5)	2	0.778
Steroid use for chronic condition, No. (%)			14 (1.1)	13 (1.2)	0 (0.0)	0 (0.0)	1 (2.4)	3	0.319
Bleeding disorders, No. (%)			12 (0.9)	8 (0.7)	4 (2.4)	0 (0.0)	0 (0.0)	4	0.181
Systemic Sepsis, No. (%)			2 (0.2)	1 (0.1)	1 (0.6)	0 (0.0)	0 (0.0)	5	0.358
ASA classification, No. (%)	1		363 (27.2)	288 (27)	55 (32.5)	9 (16.7)	11 (26.2)	16	0.011
	2		849 (63.7)	693 (64.9)	98 (58.0)	36 (66.7)	22 (52.4)		
	3		121 (9.1)	87 (8.1)_a_	16 (9.5)_a,b_	9 (16.7)_a,b_	9 (21.4)_b_		

Postoperative complications

Table 3 compares the incidence of complications across hysterectomy routes. The 30-day incidence of any complication following transmasculine hysterectomy was 6.5% (N=86/1333). This incidence was similar among hysterectomy routes. The most common complications were surgical site infection and unplanned reoperation (N=25 (1.9%), each). The incidence of postoperative complications, as well as any postoperative complication, was not significantly different across increasing BMI categories.

**Table 3:** Frequency of postoperative complications compared across the different routes of hysterectomy. Acronyms – LH: laparoscopic hysterectomy; LAVH: laparoscopic-assisted vaginal hysterectomy; VH: vaginal hysterectomy; AH: abdominal hysterectomy. Fisher’s exact test is used to compare proportions. Data is presented as No. (%). The subset of patient categories whose proportions significantly differ from each other (P <0.05) have different subscript letters, a and b. P <0.05 is considered statistically significant.

Demographics and surgical characteristics	Overall (N=1333)	LH (N=1068)	LAVH (N=169)	VH (N=54)	AH (N=42)	Test statistic	P
At least one complication	86 (6.5)	66 (6.2)	13 (7.7)	2 (3.7)	5 (11.9)	3	0.340
Unplanned reoperation	25 (1.9)	19 (1.8)	3 (1.8)	1 (1.9)	2 (4.8)	2	0.392
Unplanned readmission	24 (1.8)	19 (1.8)	5 (3.0)	0 (0.0)	0 (0.0)	2	0.560
Unplanned intubation	4 (0.3)	1 (0.1)_a_	3 (1.8)_b_	0 (0.0)_a,b_	0 (0.0)_a,b_	9	0.037
Mortality	2 (0.2)	1 (0.1)	1 (0.6)	0 (0.0)	0 (0.0)	5	0.358
Surgical site infection	25 (1.9)	18 (1.7)	5 (3.0)	1 (1.9)	1 (2.4)	2	0.438
Wound disruption	4 (0.3)	3 (0.3)	1 (0.6)	0 (0.0)	0 (0.0)	2	0.588
Pneumonia	2 (0.2)	2 (0.2)	0 (0.0)	0 (0.0)	0 (0.0)	2	1.000
Pulmonary embolism	1 (0.1)	0 (0.0)	1 (0.6)	0 (0.0)	0 (0.0)	8	0.199
Ventilator > 48 hours	1 (0.1)	0 (0.0)	1 (0.6)	0 (0.0)	0 (0.0)	8	0.199
Urinary tract infection	24 (1.8)	19 (1.8)	1 (0.6)	1 (1.9)	3 (7.1)	6	0.066
Bleeding requiring transfusions	3 (0.2)	0 (0.0)_a_	1 (0.6)_a,b_	0 (0.0)_a,b_	2 (4.8)_b_	16	<0.001
Sepsis	2 (0.2)	2 (0.2)	0 (0.0)	0 (0.0)	0 (0.0)	2	1.000
Septic shock	1 (0.1)	0 (0.0)	1 (0.6)	0 (0.0)	0 (0.0)	8	0.199
Hospital stay > 30 days	1 (0.1)	1 (0.1)	0 (0.0)	0 (0.0)	0 (0.0)	4	1.000
Clostridium difficle infection	1 (0.1)	1 (0.1)	0 (0.0)	0 (0.0)	0 (0.0)	4	1.000

LAVH was associated with a significantly higher incidence of unplanned intubation compared with LH, VH, and AH (N=3 (1.8%), N=1 (0.1%), N=0 (0.0%), N=0 (0.0%), respectively; P=0.037). After controlling for baseline comorbidities and ASA classification, LAVH was still associated with significantly greater odds of unplanned intubation compared with LH (adjusted odds ratio (aOR) 95% confidence interval (CI) 28.1 (2.3->100)).

AH was associated with significantly higher incidence of bleeding requiring transfusion compared with LAVH, LH, and VH (N=2 (4.8%), N=1 (0.6%), N=0 (0.0%), N=0 (0.0%), respectively; P<0.001). After controlling for baseline comorbidities and ASA classification, AH was no longer associated with greater odds of perioperative bleeding requiring transfusion compared with LH.

## Discussion

Principle findings

In this retrospective study, the first of its kind with the largest sample size, data from ACS-NSQIP were used to compare postoperative complications between the different hysterectomy routes among transgender men. LH was the most common hysterectomy route in this population. The AH group had a significantly higher proportion of patients with ASA class 3 when compared to LH, a significantly higher average BMI, and a significantly higher length of stay when compared to other routes. The incidence of any 30-day complication following transmasculine hysterectomy was 6.5% (N=86/1333) and was similar across all four routes, with surgical site infection and unplanned reoperation being the most common. LAVH was associated with greater odds of unplanned intubation compared to other routes even after controlling for baseline comorbidities and ASA classification.

Results in the context of what is known

Our 6.5% incidence of 30-day complications following hysterectomy in 1333 transgender men is slightly higher than what has been reported in recent literature (3-4%) [7,14], though it still suggests that this is a comparably safe procedure in this population. This difference may reflect our study’s larger and more diverse patient cohort, which likely includes a broader range of clinical scenarios, comorbidities, and surgical techniques. Unlike earlier studies that were limited by smaller sample sizes and potential selection bias toward healthier individuals or more uniform surgical settings, our dataset may better represent real-world surgical outcomes across multiple institutions. Additionally, the inclusion of various hysterectomy approaches, rather than limiting analysis to a single technique, may contribute to a more comprehensive, albeit slightly higher, complication frequency. Notably, existing literature does not indicate any significant differences in post-hysterectomy complications between transgender men and their cisgender women counterparts. In a 2018 NSQIP study that reviewed 30-day post-hysterectomy complications among 159,736 hysterectomies performed (521 in transgender men), the incidence of postoperative complications after hysterectomy in transgender men was found to be similar to the incidence observed in cisgender women (3.4% vs 3.3%, P=0.92), and transgender man status was not a predictor of post-hysterectomy complications [14]. Similar findings were reported by another NSQIP study in 2020 on 89 transgender men comparing post-operative complications of hysterectomy procedures in transgender men to hysterectomies for menstrual disorders [7]. In a more recent study, the incidence of vaginal cuff dehiscence following minimally invasive hysterectomy was found to be 6.1% (N=3/49) in transgender men undergoing minimally invasive hysterectomy for gender-affirmation, versus an incidence of 1.7% (N=2/117) in cisgender patients undergoing the same procedure for other indications. This was not statistically significant though due to a relatively small sample size [15]. Our study is unique as it represents the largest sample size to date of transgender men and is the first to compare different hysterectomy approaches in this patient population, specifically analyzing postoperative complications.

Our finding that the average length of stay for AH is significantly longer than that of other routes corroborates that of studies in cisgender females [8]. However, studies in cisgender women also suggest that the abdominal approach is associated with a higher risk of wound infection, incisional hernia, and longer hospital stays when compared to minimally invasive (vaginal and laparoscopic) approaches, making the latter a preferred option when feasible [9-11]. In one study, Bretschneider et al. found that minimally invasive approaches were associated with a lower risk of complications across both cisgender women and transgender men [14]. Conversely, we did not find any statistically significant differences in the incidence of 30-day complications among the four different routes of hysterectomy in our cohort of transgender men. Our results further imply that vaginal hysterectomy is a safe procedure for transgender men, as it is not associated with a higher risk of adverse complications compared to other routes.

The increased risk of bleeding requiring transfusion observed in patients undergoing AH compared with those undergoing laparoscopic approaches may be influenced by the underlying medical complexity of these patients. For example, transgender men treated with AH were more likely to have ASA class 3 and higher BMI compared with transgender men treated with other approaches. Moreover, individuals selected for AH are often those with larger uterine masses, extensive pelvic pathology such as severe endometriosis or adhesions, or a suspected gynecologic malignancy [13]. These factors make them unsuitable candidates for less invasive techniques like LH and inherently increase their risk for perioperative complications. The need for a more extensive surgical procedure in AH, often involving larger incisions and more extensive tissue dissection, may further contribute to the elevated risk of bleeding and other complications. This highlights the importance of patient selection and underscores that the higher complication rate in AH may reflect the complexity of the cases rather than the inherent risk of the surgical approach itself. Our findings also suggest that patient-specific factors such as BMI and ASA classification continue to play an important role in route selection for hysterectomy in transgender men.

Clinical implications

Specific routes of hysterectomy come with distinct considerations. While there is little data on such a link in cisgender women, our findings suggest a potential association between LAVH and unplanned intubation in this cohort of transgender men, even after adjusting for comorbidities. Careful patient selection and consideration of individual factors are focal in determining the most appropriate hysterectomy approach for transgender men.

Research implications

Moving forward, further studies, such as randomized trials, are warranted to explore long-term outcomes and patient-reported experiences to address care gaps and inform practice standards in transgender men seeking hysterectomy. These future studies can contribute to enhancing the quality of care and surgical decision-making for this underserved population by incorporating a more comprehensive approach that considers both clinical outcomes and patient perspectives.

Strengths and limitations

Our study is not without limitations. Due to the nature of the NSQIP database, complication records are confined to a 30-day postoperative tracking period. Focusing solely on complications within this timeframe may not encompass longer-term outcomes that could be pertinent to the study. Moreover, the NSQIP database does not include qualitative data related to patient experience, the psychosocial impact of hysterectomy on transgender men, or socioeconomic variables that might influence outcomes, such as access to healthcare, socioeconomic status, and insurance coverage. This information, if available, could play a critical role in understanding the surgical planning and decision-making process between physicians and patients. Finally, the study's exclusion of certain types of hysterectomies (e.g., those involving lymphadenectomy or due to malignancy) may restrict the scope of the research, potentially affecting the applicability of the findings to all cases of hysterectomy. 

A key strength of the study is its use of a large, nationally representative, and diverse sample. It is the first study to conduct a comparative analysis of postoperative outcomes across different hysterectomy routes in transgender men, addressing a significant gap in existing literature. Our findings support the overall safety of hysterectomy in transgender men, highlighting the importance of individualized surgical planning that considers patient characteristics and procedural risks to guide hysterectomy route selection and optimize outcomes.

## Conclusions

The study’s results suggest that hysterectomy is a comparably safe procedure for transgender men, with a 30-day complication incidence of 6.5% (N=86/1333) due primarily to surgical site infection and unplanned reoperation. There were no significant differences in the 30-day complication rates across the four routes examined: VH, LH, LAVH, and AH. However, certain procedure-specific considerations emerged, such as AH being associated with a longer hospital stay and LAVH showing a higher incidence of unplanned intubation, even after adjusting for baseline comorbidities.
